# Spirometric variability in smokers: transitions in COPD diagnosis in a five-year longitudinal study

**DOI:** 10.1186/s12931-016-0468-7

**Published:** 2016-11-10

**Authors:** Akshay Sood, Hans Petersen, Clifford Qualls, Paula M. Meek, Rodrigo Vazquez-Guillamet, Bartolome R. Celli, Yohannes Tesfaigzi

**Affiliations:** 1 Department of Internal Medicine, University of New Mexico School of Medicine, Albuquerque, NM USA; 2 COPD program, Lovelace Respiratory Research Institute, Albuquerque, NM USA; 3 Office of Research, University of New Mexico Health Sciences Ctr, Albuquerque, NM USA; 4 University of Colorado College of Nursing, Denver, CO USA; 5 Department of Internal Medicine, Brigham and Women’s Hospital, Harvard Medical School, Boston, MA USA

## Abstract

**Background:**

Spirometrically-defined chronic obstructive pulmonary disease (COPD) is considered progressive but its natural history is inadequately studied. We hypothesized that spirometrically-defined COPD states could undergo beneficial transitions.

**Methods:**

Participants in the Lovelace Smokers’ Cohort (*n* = 1553), primarily women, were longitudinally studied over 5 years. Spirometric states included normal postbronchodilator spirometry, COPD Stage I, Unclassified state, and COPD Stage II+, as defined by GOLD guidelines. Beneficial transitions included either a decrease in disease severity, including resolution of spirometric abnormality, or maintenance of non-diseased state. ‘All smokers’ (*n* = 1553) and subgroups with normal and abnormal spirometry at baseline (*n* = 956 and 597 respectively) were separately analyzed. Markov-like model of transition probabilities over an average follow-up period of 5 years were calculated.

**Results:**

Among ‘all smokers’, COPD Stage I, Unclassified, and COPD Stage II+ states were associated with probabilities of 16, 39, and 22 % respectively for beneficial transitions, and of 16, 35, and 4 % respectively for resolution. Beneficial transitions were more common for new-onset disease than for pre-existing disease (*p* < 0.001). Beneficial transitions were less common among older smokers, men, or those with bronchial hyperresponsiveness but more common among Hispanics and smokers with excess weight.

**Conclusions:**

This observational study of ever smokers, shows that spirometrically-defined COPD states, may not be uniformly progressive and can improve or resolve over time. The implication of these findings is that the spirometric diagnosis of COPD can be unstable. Furthermore, COPD may have a pre-disease state when interventions might help reverse or change its natural history.

**Trial registration:**

NA.

**Electronic supplementary material:**

The online version of this article (doi:10.1186/s12931-016-0468-7) contains supplementary material, which is available to authorized users.

## Background

Chronic obstructive pulmonary disease (COPD) is a leading cause of death in the United States and worldwide [1, 2]. COPD, defined when a certain spirometric threshold of airflow obstruction is met, is generally considered to be progressive, but its natural history is not well established. Describing spirometric variability in smokers in 1977, Fletcher and Peto suggested that non-susceptible smokers may stay in a normal spirometric state over time [3]. Further, susceptible smokers, who quit smoking, may experience a subsequent rate of decline of forced expiratory volume in one second (FEV_1_), comparable to that of non-smokers [3]. However, the longitudinal stability of the diagnosis provided by cross-sectional spirometric exam in smokers at risk for mild obstruction, is not known.

Chronic diseases may change their state-by either progressing or regressing. Changes of state i.e. transitions, can be observed longitudinally. By studying four discrete spirometric states as defined by the Global Initiative for Chronic Obstructive Lung Disease (GOLD) guidelines [4] (smokers with normal spirometry, GOLD unclassified state, COPD GOLD stage I, and COPD GOLD stage II or greater), we can examine the transitions between these states over time. Transition probabilities, are the likelihood of transitioning between states over a fixed unit of time (e.g. the time between scheduled examination visits in a study). The ‘operational’ definition of ‘beneficial transitions’ in this study, includes both true beneficial transitions and successful primary prevention of COPD. True beneficial transitions occur when disease states improve to a less severe state (including to a non-diseased or healthy state, which is also referred to as resolution of disease). Although strictly not a transition, but still beneficial, successful primary prevention of disease occurs if the non-diseased state is maintained during the unit of time of follow-up. Multistate Markov-like models have been previously used to study the natural history of several chronic diseases that have a natural interpretation in terms of staged progression [5–7]. This approach has not been previously used to study COPD, which is currently a gap in the literature. The Methods section in the Additional file 1, addresses additional concepts regarding this analytical approach.

This study tested the hypothesis that COPD states could undergo beneficial transitions. To this end, we determined the longitudinal stability of the spirometrically-defined diagnosis of COPD states in smokers. Beneficial transitions probabilities were examined over discrete 18-month units of observation, over a mean period of 5 years per subject, in a cohort of largely women smokers, without any organized intervention. We also determined the predictors that affected beneficial transitions. Primary analysis included ‘all smokers’, irrespective of their spirometric disease status at baseline (*n* = 1553). Secondary analyses were performed in subgroups of ‘smokers with normal spirometry at baseline’ (*n* = 946) to longitudinally study new-onset or incident disease, and ‘smokers with abnormal spirometry at baseline’ (*n* = 597) to longitudinally study pre-existing disease.

## Methods

### Study design, setting and population

This longitudinal, observational, epidemiological study, included 1553 smokers (approximately 80 % women) who participated in the Lovelace Smokers’ Cohort at Albuquerque, New Mexico, U.S.A. (see Additional file 1: Figure SE3). Since women are underrepresented in most studies of airflow obstruction, this large cohort of women, ever-smokers, was initially assembled to study the susceptibility of women to the adverse effects of cigarette smoking, but later added men ever-smokers [8]. The study began enrolling participants in March 2001. The catchment area for this cohort was Albuquerque and its surrounding communities, comprising a diverse population of approximately 700,000 persons at an elevation of over 5000 ft, with a stable outdoor air quality during the study period. Most participants were recruited from community-dwelling smokers through newspaper or television advertisements and were paid a small stipend for their participation. Subjects were followed at 18-month intervals. Details regarding this cohort have been previously published [9–11].

### Eligibility criteria

Participants were aged 40 to 75 years, former or current smokers, with a minimum smoking history of 20 pack-years on initial screening, and able to understand English. Participants with at least two postbronchodilator spirometry tests performed 18 months apart were included (*n* = 1553).

### Study measurements

All tests were conducted at Lovelace Scientific Resources (Albuquerque, NM). Information related to demographics, respiratory diseases, medications, and smoking was obtained by self-report from all study participants *via* standard questionnaires, by trained personnel, at each visit. Height (without shoes) and weight was measured at each visit using standard criteria and body mass index (BMI) was calculated.

An average of four pre- and post-bronchodilator spirometry tests were performed on each subject, at baseline and at 18 month intervals, over a mean period of 5 years, strictly adhering to the American Thoracic Society (ATS) guidelines [12]. An increase in value by ≥ 12 % and 200 mL compared with baseline in FEV1 and/or FVC during a single testing session, was used to define significant bronchodilator reversibility [13]. Respiratory therapists were monitored and periodically re-credentialed, as part of a standardized laboratory proficiency testing plan. An independent audit of computer-generated error codes by an investigator not involved with the collection of spirometric data, revealed that >95 % of spirometry tests met the 2005 ATS guidelines for test quality [14]. Expiratory time for the spirometric maneuvers was however not included as a covariate due to incomplete data availability. Additional details are provided in the Additional file 1.

### Outcomes

Spirometric states were defined based upon the GOLD criteria postbronchodilator FEV_1_/FVC ratio [4] and percent predicted FEV_1_ value using the third National Health and Nutrition Examination Survey or NHANES-III reference equations [15], shown in Table 1. GOLD Unclassified state is usually described as a restrictive spirometric pattern, although ‘unclassified’ or ‘nonspecific’ spirometry and ‘preserved ratio impaired spirometry (PRISm)’ terms, are also used by some investigators [16–18]. In an alternative analysis presented in Additional file 1: Table SE2, COPD states were described by statistically-defined NHANES III lower limit of normal for the FEV_1_/FVC ratio. Health status, as defined by the St. George’s Respiratory Questionnaire (SGRQ), [19] and post-bronchodilator FEV_1_ values were together used to classify the ordinal severity of spirometric states, as shown in Table 2. The four discrete spirometric states were longitudinally followed, and the change in rank order for these states was used to study beneficial transitions.

**Table 1:** Definition of Spirometric States, Albuquerque, New Mexico, 2001–2015, Lovelace Smokers’ Cohort

	Smokers with normal spirometry	COPD GOLD stage I	GOLD Unclassified	COPD GOLD stage II+
Postbronchodilator FEV1/FVC ratio^a^	≥0.70	<0.70	≥0.70	<0.70
Postbronchodilator FEV_1_ percent predicted^a^	≥80 %	≥80 %	<80 %	<80 %

*Abbreviations*: *COPD* Chronic Obstructive Pulmonary Disease, *FEV*_*1*_ forced expiratory volume in one second, *FVC* forced vital capacity, *GOLD* Global Initiative for Chronic Obstructive Lung Disease

^a^Postbronchodilator FEV_1_/FVC ratio was based on the GOLD criteria [4] and percent predicted FEV_1_ value was defined by the third National Health and Nutrition Examination Survey or NHANES-III reference equations [15])

**Table 2:** Characteristics Used for Ordinal Ranking of Spirometrically-defined States at Baseline Examination Visit, Among ‘All Smokers’, Albuquerque, New Mexico, 2001–2015, Lovelace Smokers’ Cohort

Characteristic	Smokers with normal spirometry (*n* = 956)	COPD GOLD stage I (*n* = 145)	GOLD Unclassified (*n* = 191)	COPD GOLD stage II+ (*n* = 261)	*P* value
SGRQ total score	17.0 ± 15.2	17.9 ± 13.9	25.6 ± 19.2	31.6 ± 19.8	<0.001
SGRQ symptom subscale score	25.6 ± 21.1	29.9 ± 21.7	33.1 ± 23.1	41.6 ± 24.4	<0.001
SGRQ activity subscale score	25.6 ± 22.7	25.0 ± 21	37.3 ± 26.3	43.8 ± 25.7	<0.001
SGRQ impact subscale score	8.5 ± 11.9	8.7 ± 9.8	14.8 ± 16.4	18.7 ± 16.7	<0.001
FEV_1_ (in liters)	2.8 ± 0.6	2.7 ± 0.7	2.1 ± 0.5	1.7 ± 0.6	<0.001
FEV_1_ percent predicted	97.4 ± 10.6	91.0 ± 8.9	72.0 ± 8.5	61.2 ± 14.7	<0.001

*Abbreviations*: *COPD* Chronic Obstructive Pulmonary Disease, *FEV*_*1*_ forced expiratory volume in one second, *FVC* forced vital capacity, *GOLD* Global Initiative for Chronic Obstructive Lung Disease, *SGRQ* St. George Respiratory Questionnaire

**Fig. 1:**
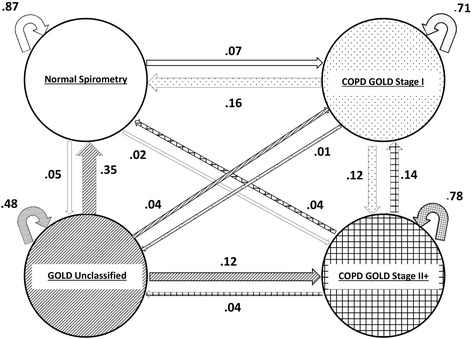
Multi-state Markov-like model analyzing longitudinal transition probabilities between spirometric states, among ‘all smokers’, irrespective of spirometric disease at baseline, over approximately 5 years, Albuquerque, New Mexico, 2001–2015, Lovelace Smokers’ Cohort. Curved block arrows represents transition probabilities of staying within the same spirometric state between study examination visits at 18 month intervals. Straight arrows represent transition probabilities of change in spirometric state between study examination visits. Strength of transition probabilities is represented by the width of the straight arrow. Transition probabilities for each spirometric state are depicted in the same color as the sphere representing the spirometric state

A beneficial transition, our primary outcome variable, was operationally defined by either a decrease in spirometric disease state severity (i.e. true beneficial transition) or continued maintenance of normal spirometric state (i.e. primary prevention of disease) at any time during longitudinal follow-up. For example (as shown in Fig. 1), a beneficial transition for the GOLD Unclassified state would be to improve to either normal spirometry or GOLD Stage 1 state at any time during longitudinal follow-up. On the other hand, for the normal spirometric state, a beneficial transition would be defined by longitudinal stability in the normal state. Resolution of disease state, the secondary outcome variable, was defined by change of spirometrically-defined disease state, to a normal spirometry state, at any time during longitudinal follow-up. In Fig. 1, this would be illustrated by a line from any of the spirometric GOLD states to normal spirometry state.

### Statistical analysis

Summary statistics included means, standard deviations (S.D.), medians, and interquartile ranges for the continuous variables and proportions for the categorical variables. Chi-square and Fisher’s exact tests were used for the bivariate analysis of categorical variables, while the two-sample *t*-test was used for continuous variables. Analysis of variance was used to compare characteristics among the spirometric states. Multi-state Markov-like model of transition probabilities over an average follow-up period of 5 years were calculated. Candidate predictors for beneficial transitions, as described in the Methods section in the Additional file 1, were analyzed in univariate and multivariable analyses using the general linear model. Interactions between baseline spirometric state and candidate predictor variables on beneficial transitions were analyzed using formal tests of interaction. To exclude possible selection bias from loss to follow-up after two initial visits, sensitivity analyses were additionally performed by excluding those with loss to follow-up (data not presented). All analyses were conducted in Statistical Analysis Software SAS 9.4 (Cary, NC). A two-sided *p*-value of < 0.05 was considered statistically significant. Informed consent was obtained from all study participants. This study was approved by the Western Institutional Review Board (No. 20031684).

## Results

As shown in Table 3 and Additional file 1: Table SE1, study participants were mostly middle-aged, overweight, women, who were current and heavy smokers. Health status (including symptoms, activity limitation, and disease impact) and post-bronchodilator FEV_1_ values were progressively and significantly (*p* < 0.001 for all analyses) worse from smokers with normal spirometry, to COPD Stage I, to Unclassified state, to COPD Stage II+ (Table 2).

**Table 3:** Differences in Characteristics Between Ordinally-ranked Spirometrically-defined States at Baseline Examination Visit, Among ‘All Smokers’, Albuquerque, New Mexico, 2001–2015, Lovelace Smokers’ Cohort

Characteristic	Smokers with normal spirometry (*n* = 956)	COPD GOLD stage I (*n* = 145)	GOLD Unclassified (*n* = 191)	COPD GOLD stage II+ (*n* = 261)	*P* value
Age (in years)	54.0 ± 9.1	61.0 ± 9.7	56.1 ± 8.4	61.0 ± 8.6	<0.001
Men (%)	18.1 %	43.5 %	18.9 %	24.5 %	0.001
Hispanic ethnicity (%)	20.3 %	8.3 %	22.5 %	6.5 %	<0.001
BMI (in kg/m^2^)	28.1 ± 5.5	26.5 ± 5	31.2 ± 8.5	27.0 ± 6.1	0.86
Self-reported history of provider-diagnosed hypertension (%)	27.9 %	37.9 %	48.4 %	37.3 %	<0.001
Current smoking (%)	54.0 %	51.7 %	64.4 %	51.0 %	0.75
Pack-years of smoking	35.7 ± 17.8	47.4 ± 23	38.9 ± 19	50.4 ± 24.5	<0.001
Diabetes (%)	6.5 %	5.5 %	14.2 %	5.4 %	0.33
Postbronchodilator FVC (in liters)	3.6 ± 0.8	4.1 ± 1.1	2.7 ± 0.6	3.0 ± 0.9	<0.001
Postbronchodilator FEV_1_/FVC (%)	78.7 ± 4.5	65.4 ± 3.4	76.4 ± 4.4	56.4 ± 10.1	<0.001
Postbronchodilator FVC percent predicted	97.5 ± 10.7	106.9 ± 11.7	73.8 ± 9.4	83.3 ± 15.2	<0.001
Postbronchodilator FEV_1_/FVC (%)	78.7 ± 4.5	65.4 ± 3.4	76.4 ± 4.4	56.4 ± 10.1	<0.001
Self-reported history of provider-diagnosed asthma	13.7 %	20 %	23.2 %	30.4 %	<0.001
Presence of significant bronchodilator reversibility (%)	1.8 %	9.7 %	5.8 %	13.8 %	<0.001
History of provider-diagnosed asthma + bronchodilator reversibility (%)	0.2 %	2.1 %	3.1 %	4.6 %	<0.001
Follow-up period (in years)	5.1 ± 2.5	4.6 ± 2.3	5.0 ± 2.6	4.7 ± 2.5	0.03

*Abbreviations*: *BMI* body mass index, *COPD* Chronic Obstructive Pulmonary Disease, *FEV*_*1*_ forced expiratory volume in one second, *FVC* forced vital capacity, *GOLD* Global Initiative for Chronic Obstructive Lung Disease, *SGRQ* St. George Respiratory Questionnaire

Beneficial transitions of spirometric disease states:

When ‘all smokers’ were followed for 18-month units of time over a mean period of follow-up of 5 years, the probabilities for any longitudinal beneficial transition were 16, 39, and 22 % for COPD Stage I, GOLD Unclassified, and COPD Stage II+, respectively (Fig. 1 and Table 4). The corresponding probabilities for resolution of spirometric abnormality were 16, 35, and 4 % respectively. When comparison was made between ‘smokers with normal spirometry at baseline’ (at risk for new-onset disease) vs. ‘smokers with abnormal spirometry at baseline’ (with pre-existing disease), the probabilities for any longitudinal beneficial transition or for resolution during study follow-up were more common for new-onset disease than for pre-existing disease (*P* < 0.001 for both analyses; Additional file 1: Figures SE1 and SE2; and Table 4). Data on harmful transitions (defined by either an increase in spirometric state severity or continued maintenance of COPD GOLD Stage II+ state) are also provided in Additional file 1: Table SE4 and can be visually discerned from Fig. 1 and Additional file 1: Figures SE1 and SE2.

**Table 4:** Summary of Beneficial Transition Probabilities for Spirometrically-defined States at Any Time Over Approximately 5 Years, Albuquerque, New Mexico, 2001–2015, Lovelace Smokers’ Cohort

	Normal spirometry at any time	COPD GOLD Stage I at any time	GOLD Unclassified at any time	COPD GOLD Stage II+ at any time
All smokers, irrespective of spirometric disease at baseline (*n* = 1553)				
All beneficial transitions^a^	87 %	16 %	39 %	22 %
Resolution of disease^a^	NA	16 %	35 %	4 %
Smokers with normal spirometry at baseline (*n* = 956)*				
All beneficial transitions^a^	89 %	26 %	64 %	64 %
Resolution of disease^a^	NA	26 %	57 %	29 %
Smokers with abnormal spirometry at baseline (*n* = 597)				
All beneficial transitions^a^	55 %	12 %	33 %	20 %
Resolution of disease^a^	NA	12 %	29 %	2 %

*Abbreviations COPD* Chronic Obstructive Pulmonary Disease, *GOLD* Global Initiative for Chronic Obstructive Lung Disease

^a^A beneficial transition, our primary outcome variable, was defined by either a decrease in spirometric state severity, including resolution, or continued maintenance of normal spirometric state at any time during longitudinal follow-up. Resolution of disease state, our secondary outcome variable, was defined by change of spirometrically-defined diseased states to normal spirometry state at any time during longitudinal follow-up

*reflect transitions for new onset disease

Additional data are provided in Fig. 1 in the main text and Additional file 1: Figures SE1 and SE2, including data on change in severity of individual spirometric disease states

Probabilities for beneficial transition and for resolution of disease were significantly different between groups with ‘normal’ and ‘abnormal spirometry at baseline’ (*P* < 0.001 for both analyses), using SAS Proc GENMOD

A similar table using the statistically defined NHANES-III lower limits of normal for FEV_1_/FVC ratio to define obstruction is presented in the Additional file 1: Table SE2

### Predictors for beneficial transitions

The individual spirometric state at the onset of the 18-month observation unit was a significant predictor of beneficial transitions (Table 5). GOLD Unclassified was the most likely state to demonstrate either a beneficial transition or resolution of disease (Table 4).

**Table 5:** Baseline Predictors of Beneficial Transitions Over Approximately 5 Years, Albuquerque, New Mexico, 2001–2015, Lovelace Smokers’ Cohort

Characteristic	Univariate analysis	Multivariable analysis^a^
	Odds Ratio (95 % CI)	Odds Ratio (95 % CI)
	‘All Smokers’, irrespective of spirometric disease at baseline	
	*n* = 1553	
Abnormal spirometric state at onset of 18-month observation unit	0.33 (0.31, 0.36)**	0.36 (0.33, 0.39)**
Age ≥ 60 year.	0.67 (0.56, 0.79)**	0.69 (0.56, 0.85)**
Male sex	0.50 (0.43, 0.58)**	0.51 (0.43, 0.6)**
Hispanic ethnicity	1.70 (1.44, 2.00)**	1.90 (1.47, 2.46)**
Excess weight (BMI ≥ 25 kg/m2)	1.26 (1.1, 1.45)**	1.38 (1.16, 1.64)**
History of hypertension	0.70 (0.61, 0.82)**	0.88 (0.74, 1.06)
History of asthma + bronchodilator reversibility	0.23 (0.11, 0.52)**	0.42 (0.23, 0.77)**
	‘Smokers with normal spirometry at baseline’	
	*n* = 956	
Abnormal spirometric state at onset of 18-month observation unit	0.34 (0.27, 0.42)**	0.34 (0.28, 0.42)**
Age ≥ 60 year.	0.79 (0.67, 0.94)**	0.88 (0.63, 1.25)
Male sex	0.78 (0.67, 0.91)**	0.51 (0.39, 0.67)**
Hispanic ethnicity	1.17 (0.99, 1.38)	1.62 (1.09, 2.41)*
Excess weight (BMI ≥ 25 kg/m2)	1.21 (1.05, 1.38)**	1.61 (1.22, 2.11)**
History of hypertension	0.92 (0.79, 1.07)	1.03 (0.77, 1.38)
History of asthma + bronchodilator reversibility	0.19 (0.03, 1.21)	0.12 (0, 2.77)
	‘Smokers with abnormal spirometry at baseline’	
	*n* = 597	
Abnormal spirometric state at onset of 18-month observation unit	0.77 (0.67, 0.88)**	0.81 (0.7, 0.93)**
Age ≥ 60 year.	0.86 (0.63, 1.16)	0.89 (0.64, 1.23)
Male sex	0.57 (0.44, 0.75)**	0.57 (0.43, 0.75)**
Hispanic ethnicity	2.46 (1.74, 3.49)**	2.59 (1.78, 3.78)**
Excess weight (BMI ≥ 25 kg/m2)	1.34 (1.03, 1.75)*	1.14 (0.85, 1.52)
History of hypertension	0.96 (0.73, 1.25)	1.07 (0.8, 1.42)
History of asthma + bronchodilator reversibility	0.89 (0.39, 2.06)	0.76 (0.37, 1.59)

*Abbreviations*: *BMI* body mass index

^a^In the multivariable analysis, each predictor in the model was adjusted for all the remaining predictors in the model

*P* values <0.05 and 0.01 are represented by symbols * and ** respectively

Repeated measures of smoking status, evaluated as a time-varying covariate, did not predict beneficial transition (*p* = 0.38) and was therefore not included as a covariate in the multivariable analyses

Older smokers (age ≥ 60 years), men, and non-Hispanic whites were less likely to experience beneficial transitions than younger smokers, women, and Hispanics respectively. Excess weight (i.e. overweight or obese) smokers were more likely to experience beneficial transitions than normal weight smokers, but this association was not significant in the multivariable analyses in the subgroup of ‘smokers with abnormal spirometry at baseline’. Smokers with bronchial hyperresponsiveness (i.e. history of provider-diagnosed asthma plus significant bronchodilator reversibility) were less likely to experience beneficial transitions than those without bronchial hyperresponsiveness, but this association was not significant in the two subgroups of smokers. Repeated measures of smoking status, evaluated as a time-varying covariate, did not predict beneficial transition (*p* = 0.38) and was therefore not included as a covariate in the multivariable analyses in Table 5.

No significant interactions were noted between spirometric state and each of the above-mentioned predictors on beneficial transitions, implying that these predictors have non-differential, uniform effects on the various spirometric states.

Subjects lost to follow-up after two initial visits were younger in age, more likely to be current smokers, and had lower lung function values at baseline visit, than those who were not lost to follow-up. Additional detail on characteristics associated with loss to follow-up is provided in the Additional file 1: Table SE3. Analyses were repeated excluding loss to follow-up and similar results were noted as described above.

## Discussion

This observational study of ever smokers, shows that spirometrically-defined COPD states may not be uniformly progressive, and can improve or even resolve over time, without any organized intervention. Beneficial transitions are more common with new-onset disease, as compared to pre-existing disease, suggesting that smokers may experience a pre-disease state. This pre-disease state may consist of lung function fluctuating in and around the definitional spirometric range of COPD, but returning to normal. Among spirometrically-defined states, beneficial transitions at 18-month intervals are more common in GOLD Unclassified than in other COPD states. In this sample, beneficial transitions were also less common among older smokers, men, or those with bronchial hyperresponsiveness but more common among Hispanics, and individuals with excess weight.

**Fig. 2:**
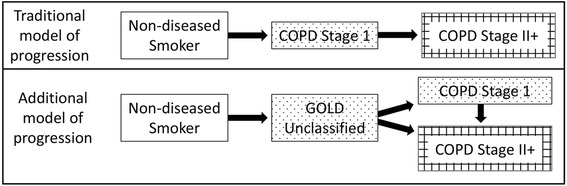
The traditional model of ‘progression’ of COPD, whereby non-diseased smokers progress to COPD GOLD Stage I and then to COPD GOLD Stage II+, is over-simplistic. Our findings suggest that not only can spirometric disease states improve or resolve over time at any point in the ‘progression’ trajectory but there may also be an additional model of disease ‘progression’ that includes GOLD Unclassified as an intermediate state between non-diseased smoker and COPD states. Abbreviations: COPD: Chronic Obstructive Pulmonary Disease; GOLD: Global Initiative for Chronic Obstructive Lung Disease

Spirometrically defined COPD is generally considered progressive, whereby individuals are expected to sequentially progress from stage I (mild COPD) through stage IV (very severe COPD) [20]. Three recent large longitudinal studies challenge this concept [21–23]. In all three studies, only a minority of patients had a rapid decline in FEV_1,_ while there was a sizeable proportion who either did not decline, or actually had an increase in FEV_1_ [21–23]. Using multi-state Markov modeling, this study supports the findings of the above-mentioned three longitudinal studies [21–23] by demonstrating that respiratory disease states, before they become well-established, have a modifiable state in which it might be possible to achieve resolution or mitigation. The study also supports an additional model of COPD progression that includes the GOLD Unclassified category as an intermediate state (Fig. 2). Individuals who demonstrate this model of COPD progression, likely have disproportionately greater small airway involvement during the early stage of disease, than individuals who follow the traditional model of progression.

Based upon these findings, it is likely that smokers experience a pre-COPD state in which their lung function fluctuates in the spirometric range of COPD, as defined by the GOLD criteria, but returns to normal. It is now understood that several chronic diseases such as hypertension and diabetes, have similar pre-disease states that are amenable to preventive and therapeutic interventions [24]. It is possible that interventions, at the level of pre-disease in COPD, might help change the natural history of COPD in smokers.

In this study, ‘all smokers’ had a 10 % likelihood of being in the GOLD Unclassified state during the course of the study, remarkably similar to the 9 % cross-sectional prevalence described in the COPDGene cohort [18]. Individuals in this state in the COPDGene cohort, are characterized by a greater BMI and prevalence of diabetes mellitus, and have significant symptoms and functional limitations [18]. Like the COPDGene cohort, we show a higher BMI value for subjects in the GOLD Unclassified state, but the difference in BMI does not completely explain their high symptom score (*P* < 0.001 after adjustment for BMI). Many GOLD Unclassified subjects in the COPDGene cohort had significant amounts of CT-assessed paracentral emphysema and evidence of small airways disease [18, 25]. In a separate longitudinal study, 15 % of GOLD Unclassified subjects developed an obstructive pattern and 3 % normalized over a median period of 3 years [26]. Another longitudinal study with an approximate 12 years of follow-up, demonstrated that 38 % of GOLD Unclassified subjects developed overt obstruction [27]. As shown in Fig. 1 and Additional file 1: Figures SE1 and SE2, 15-18 % of subjects with GOLD Unclassified state in this study, developed obstruction over a mean period of 5 years; this proportion likely increases with time. Subjects with GOLD Unclassified state are more likely to experience beneficial transitions than with any other COPD state (Table 4). We hypothesize that the inflammation of small airways that often characterizes GOLD Unclassified state, may be more amenable to preventive and therapeutic interventions than other COPD pathological changes.

The findings of this study demonstrate that excess weight increases beneficial transitions for COPD. The latter is consistent with the established literature that demonstrates a lower risk for all-cause mortality among obese patients with COPD vs. normal weight [28]. Excess weight in this study is associated with a higher FEV_1_/FVC ratio and a higher likelihood of being in the GOLD Unclassified state—the latter was the most likely state to demonstrate beneficial transition (Table 4). Older smokers, in this study, were less likely to experience beneficial transitions than younger smokers. This may reflect the differences in genetic, epigenetic or pathogenetic susceptibility to respiratory disease in younger vs. older smokers [29]. It is also possible that loss of height with aging in older smokers may lower their spirometric function [30], contributing to less frequent beneficial transitions. We however did not find a significant difference in loss of height during longitudinal follow-up in older smokers between the four spirometric states. Men were less likely than women to experience beneficial transitions. Similar findings were noted in those with bronchial hyperresponsiveness. Bronchial hyperresponsiveness, which is associated with greater COPD progression and mortality [31], may be more common among men than women in a study of emphysematous patients from the National Emphysema Trial (NETT) [32] but that is unlikely to explain the sex difference, since the findings were significant even after adjustment for bronchial hyperresponsiveness in the multivariable analysis (Table 5). Additional data from the NETT showed that men showed more emphysema and less chronic bronchitis pattern than women [33]. Since patients in the emphysematous group experience more rapid decline in lung function and higher mortality [34], the gender dimorphism in the natural history of COPD may help explain our finding. Additionally, men had greater cumulative smoking history than women (43.3 vs. 38.7 pack-years; *p* < 0.001) in the current study which may help explain the sex difference in beneficial transitions. Further, New Mexican Hispanics were more likely to experience beneficial transitions than non-Hispanic whites, despite the lower access to healthcare described among Hispanics in the literature [35]. This finding is however consistent with our previously published data that New Mexican Hispanics were less likely to have COPD and had a lower risk of rapid decline in FEV_1_ than non-Hispanic whites [9, 36]. Genetic analyses in our previously published data showed that New Mexican Hispanics have approximately one third Native American and two thirds European ancestry [9]. The Native American proportion appeared to protect against lung function decline and COPD risk [9]. These findings highlight the need for comprehensive studies in Hispanics to identify genetic factors that may be responsible for protection against COPD.

The strengths of this study relate to its large sample size, clinically-relevant study question, longitudinal nature, inclusion of large numbers of Hispanic and women populations, use of high-quality postbronchodilator spirometry tests, and the use of Multi-state Markov-like transition probabilities statistical analysis.

Limitations relate to the fact that it is unclear if beneficial transitions in spirometric state translate to beneficial longitudinal transitions in symptoms or quality of life indices. Cross-sectional data presented in Table 2 suggest that possibility. Another limitation of the study is that we did not adjust for exhalation time for the spirometry maneuver. Although the spirometry tests were performed using standard end of test criteria, it is possible that variation in exhalation duration on follow-up tests may affect corresponding FVC values, particularly for patients with airways obstruction or older subjects. However, exhalation times of ≥15 s will rarely change clinical decisions [37]. We have limited proportions of patients with severe pre-existing COPD in our cohort, who may be less likely to demonstrate beneficial transitions. This distribution is consistent with our community-based recruitment, as opposed to clinic-based recruitment. Multi-state Markov-like models attach equal weight to large changes and small changes in lung function, which is a limitation of all modeling strategies using cut scores. However, for volatile disease states, such models are an ideal analysis choice (see Additional file 1 for more detail). A transition to a normal state may represent ‘regression to the mean’, a statistical phenomenon that can make natural variation in repeated data look like a real change. Nonetheless, it is useful to report such variation, since it contradicts the accepted literature, which suggests a unidirectional progression of COPD. Further, our use of about four spirometric tests per person in this study increases the validity of our results, since multiple tests are more likely to capture true changes than two tests. Finally, since our multivariable models are below thresholds of significance, our findings cannot simply be explained by the ‘noise’ around the definitional use of cutoff scores to define transition (see Additional file 1 for more detail). We include ‘maintenance in the normal state’ within our definition of beneficial transition, since the goal of primary prevention is to prevent progression to a disease state, and such natural history is useful for future interventions (see Additional file 1 for more detail). We do however present detailed data on beneficial transitions in individual disease severity in Fig. 1 and Additional file 1: Figures SE1 and SE2 to allow the reader to differentiate ‘maintenance of normal state’ from ‘improvement’. We considered alternative analyses using survival methods that study time to first transition. However, this approach does not address subsequent transitions (which constitute the bulk of transitions) and therefore Multi-state Markov-like model is a superior approach. We may have insufficient numbers of men in this study. Few studies however have focused on COPD states in female smokers. The study also did not evaluate occupation as a predictor of transition, although this variable likely has greater value in predominantly male cohorts. The study did not exclude smokers with underlying self-reported asthma, since postbronchodilator obstruction provides a clear definition of COPD. This approach however allowed us to examine bronchial hyperresponsiveness as a predictor for beneficial transition in COPD states.

## Conclusions

This study demonstrates that the spirometric diagnosis of COPD can be unstable. Not only can airflow severity staging improve in a substantial minority but resolution of spirometric abnormalities can occur in 4–35 % of COPD states followed over 18 month interval units. This observation is based on the findings of this study in predominantly-female, community-dwelling smokers, over a follow-up period of 5 years without any organized intervention. These findings may help better describe subphenotypes of COPD when studying the underlying mechanisms and genes responsible for progression of disease. More importantly, this understanding could also lead to novel preventive and therapeutic strategies that may help prevent COPD progression or mitigate its severity, particularly during its pre-disease state before disease become established.

## Additional file

Additional file 1: Online Data Supplement. (DOCX 831 kb)

## Abbreviations

ATS: American Thoracic Society
BMI: Body mass index
COPD: Chronic Obstructive Pulmonary Disease
FEV_1_: Forced expiratory volume in one second
FVC: Forced vital capacity
GOLD: Global Initiative for Chronic Obstructive Lung Disease
ICS: Inhaled corticosteroids
LABA: Long-acting beta agonist
LAMA: Long-acting muscarinic antagonist
NHANES: National Health and Nutrition Examination Survey
SAS: Statistical analysis software
SD: Standard deviation
SGRQ: St. George Respiratory Questionnaire
