# Supplementary material for: Spirometric variability in smokers: transitions in COPD diagnosis in a five-year longitudinal study
Source: Respir Res. 2016 Nov 10;17:147. doi: 10.1186/s12931-016-0468-7 (PMC5105293; doi:10.1186/s12931-016-0468-7)
Supplement: Additional file 1: — Online Data Supplement. (DOCX 831 kb) [file 12931_2016_468_MOESM1_ESM.docx]

**ONLINE DATA SUPPLEMENT**

Title: Spirometric Variability in Smokers: Transitions in COPD Diagnosis in a Five-Year Longitudinal Study.

**Supplemental Methods:**

*Multistate Markov-like Model*

A multistate Markov-like model is used to study the natural history of COPD in this study, but this approach is not commonly used in the medical literature and therefore not readily understood. The following discussion addresses the less well understood concepts in this analytical approach.

1. The going from normal (prior state) to normal (current state) is counted as beneficial transition.

A. In the Markov chain literature, this and all other changes are called transitions. The suggestion of an illustration of a simpler model with two states may be a good suggestion; one must show all transitions otherwise the transition probabilities would not add up to 1.0; here normal-normal is one of the transitions with probability of 0.4.

Normal

Ab-

Normal

0.6

0.5

0.5

0.4

B. The normal-normal transition compared to the other possible transitions is as good as it can be; thus it is beneficial. Backing up in our point of view, the goal of primary prevention is a normal-normal transition; thus, this is the favorable outcome and should be classified as a beneficial transition.

2. Although it may be difficult for clinicians to interpret the Multi-state Markov-like model, this model is ideal to study disease transitions.

A. This problem begins with the conviction that COPD is progressive going from state to a worse state until one reaches an ‘absorbing state’ from which there is no return. This view can be quite wrong; and yet this may be the gestalt adopted by many clinicians. Perhaps we should begin by destroying this belief; after all this is a dismal prospect if clinicians had to explain it to their patient who has COPD. What probability of reversal is worth mentioning to the patient; shouldn’t these probabilities be known by clinicians?

If the disease is progressive in the way mentioned above, then standard statistical analyses can be applied to collected data. One could make the time to reach the absorbing state and conduct a survival analysis; this is often done with mortality data. Such analyses are more difficult to apply if reversals are important that make reaching an absorbing state a distant event, the more appropriate analysis would be Multi-state Markov-like modeling.

B. Understanding transitions and transition probabilities give an elevated platform to discuss disease intervention/prevention. Most think about treating the state and not the transition; these are difficult to separate. However, it is theoretically possible that a treatment eliminates a state occupancy probability (*i.e.* the current distribution of patients among the states in the model), but leaves the transition probabilities unchanged. This would be an ethereal victory; the system would soon return to its original state. That is the power of transition probabilities. More attention needs to be given to treatments that might affect transition probabilities and the mechanisms involved. Primary prevention and maybe secondary prevention are about preventing transitions. Consider a chronic disease, say diabetes mellitus, where one tracks the HbA1c level. A drug might lower the HbA1c to an acceptable level, but the patient is not considered cured. Since transition back to high levels of HbA1c is not good, the patient takes the drug regularly in order to modify (*i.e.* lower) this transition probability. So is the target treating the disease (evidently not), the high HbA1c (maybe), or the transition probability (probably)? One needs to sort out disease states, indicators/markers of disease state, and transitions among states. This will require Multi-state Markov-like modeling. Such modeling is not well represented in the literature which is a critical gap.

3. Although lack of memory assumption disqualifies a Markov model from being used, Multi-state Markov-like modeling can still be used.

It is true that our transition data do not strictly satisfy the Markov property; thus the analytical model here is a Multi-state Markov-like modeling instead of a Markov chain process. The Markov property requires the probability of the transition State j -> State i dependent on j but not on any prior history. Thus the transition probabilities in a Multi-State process are conditional probabilities that may depend on the prior transition history; the estimate of the transition probability that we provide is the (weighted) average of those conditional probabilities over the prior history:

P( i / j ) =Σ P( ϕ ) P( i / j ϕ), where ϕ represents the prior transition history.

This result is based on the Theorem of Total Probabilities. Thus, the probabilities that we compute are transition probabilities. The only concern is about projections based on repeated multiplication by the transition matrix and the resulting limiting stationary distribution among the states but we do not use these ideas in our manuscript. Therefore, our use of Multi-state Markov-like modeling is appropriate.

4. Transitions are not explained away by misclassification bias

There is no doubt that there will be misclassification of which state a subject may be in. This is also true for every diagnostic test based on a cut score used in medical practice. Viewing misclassification as noise and the true state as the signal, a diagnostic test is validated when it is shown to be correlated with the clinical outcome. If misclassification were of major effect, then the diagnostic test would not be correlated with outcome; no variable can be correlated to noise. The same is true here; since transitions are shown to be related to clinical factors, then the classification is validated and the noise of misclassification does not obscure the signal (the findings). If transition between states were entirely due to misclassification, then the transitions between these states should not be significantly related to clinical variables. To the extent that these transitions are related to clinical variables, these transitions are not explained away by misclassification bias.

*Study Measurements:*

An average of four pre- and post-bronchodilator spirometry tests were performed on each subject, at baseline and at 18 month intervals, over a mean period of 5 years. Spirometry was obtained by trained respiratory therapists strictly adhering to the 1994 update to the standard American Thoracic Society (ATS) guidelines ([1](#_ENREF_1)). After completion of baseline spirometry, all subjects were given two puffs of albuterol (90 mcg/spray metered dose inhaler) with a LiteAire® dual valve spacer (Thayer Medical Corporation, Tucson, AZ) and spirometry was repeated after 15 minutes. All patients were requested to not take any short-acting β-agonist inhalers for 4 hours prior to their appointment, unless medically necessary. Vmax Encore 22 (Viasys Respiratory Care, Yorba Linda, CA) and KoKo (Ferraris Respiratory, Louisville, CO) spirometers were used. Both machines met the standard ATS recommendations and were calibrated daily and checked at three different injection speeds, as per the ATS guidelines ([1](#_ENREF_1), [2](#_ENREF_2)). Additionally, respiratory therapists were monitored and periodically re-credentialed, as part of a standardized laboratory proficiency testing plan. An independent audit revealed that >95% of spirometry tests met the 2005 ATS guidelines ([2](#_ENREF_2)). The reference standards were those from the National Health and Nutrition Examination Survey (NHANES) III spirometric reference equations ([3](#_ENREF_3)) - the Mexican-American reference standard was used for Hispanics and the Caucasian American reference standard for non-Hispanic whites and Native Americans.

Health status was defined by the St. George Respiratory Questionnaire (SGRQ), with scores calculated for three domains (symptoms, activity, and psychosocial impacts) as well as a total score, with a higher score corresponding to lower health status ([4](#_ENREF_4)). Health status and post-bronchodilator spirometry values were used to classify the ordinal severity of spirometric states.

*Predictor variables:*

Candidate predictors of beneficial transition probabilities included age ≥ 60 years, sex, ethnicity, wood smoke exposure status, current smoking status, change in current smoking status, excess weight (BMI ≥ 25 kg/m^2^), medications (long-acting beta agonists or LABA, long-acting muscarinic antagonists or LAMA and inhaled corticosteroids or ICS), and co-morbidities (hypertension, diabetes, congestive heart failure), and bronchial hyperresponsiveness (defined as the presence of self-reported history of provider diagnosed asthma plus significant bronchodilator reversibility on spirometric testing), all measured at baseline examination visit. Predictors that were significant in univariate analyses were retained in subsequent multivariable analyses.

**Supplemental Figures**

Supplementary Figure E1.

Figure Legend E1: Multi-state Markov-like model analyzing longitudinal transition probabilities between spirometric states, among smokers with a baseline normal spirometry, over approximately 5 years, Albuquerque, New Mexico, 2001-2015, Lovelace Smokers’ Cohort.


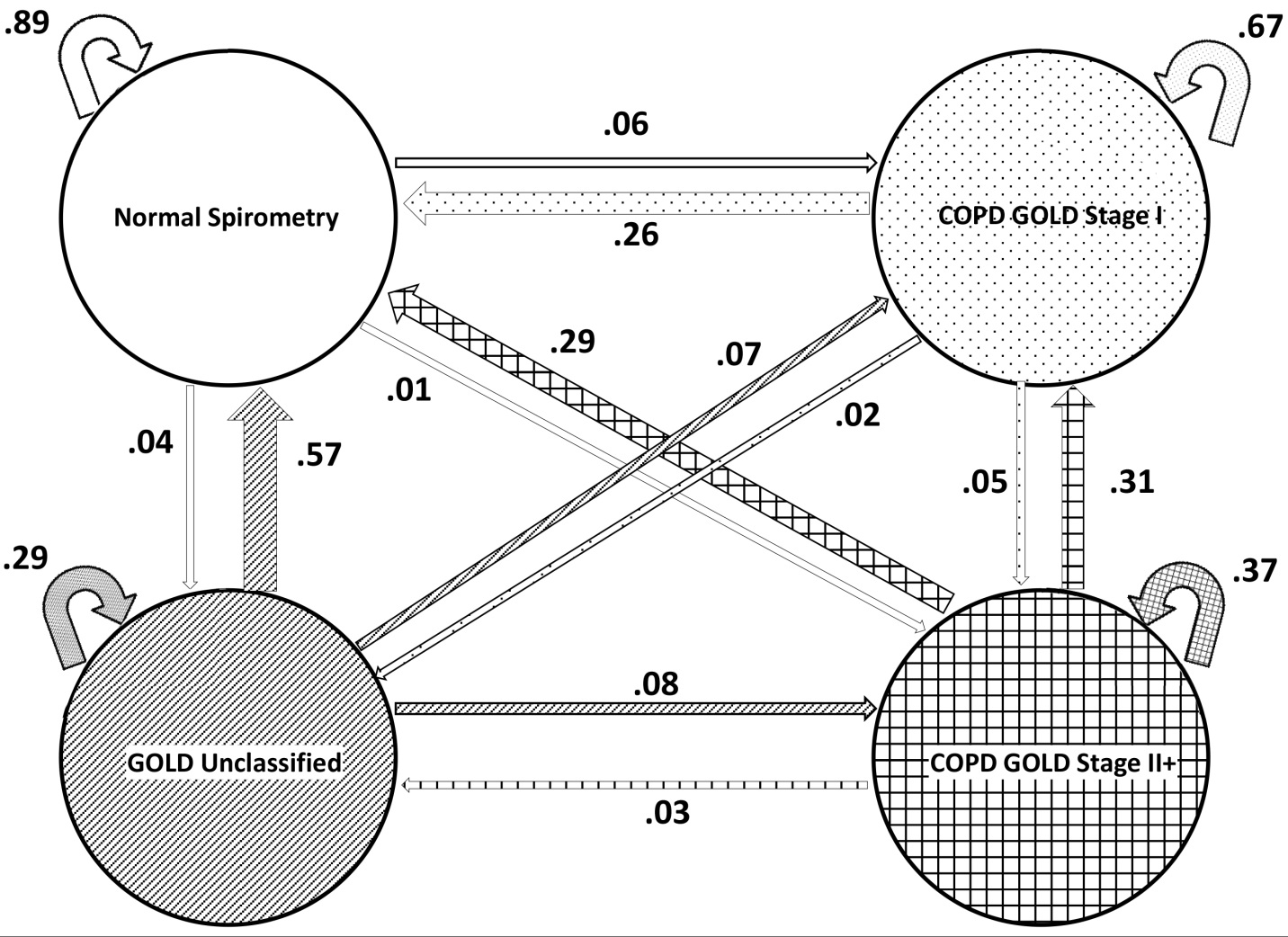


Footnote to Figure E1: Curved block arrows represents transition probabilities of staying within the same spirometric state between study examination visits at 18 month intervals. Straight arrows represent transition probabilities of change in spirometric state between study examination visits. Strength of transition probabilities is represented by the width of the straight arrow. Transition probabilities for each spirometric state are depicted in the same color as the sphere representing the spirometric state.

Supplementary Figure E2.

Figure Legend E2. Multi-state Markov-like model analyzing longitudinal transition probabilities between spirometric states, among ‘smokers with abnormal spirometry at baseline’, over approximately 5 years, Albuquerque, New Mexico, 2001-2015, Lovelace Smokers’ Cohort.


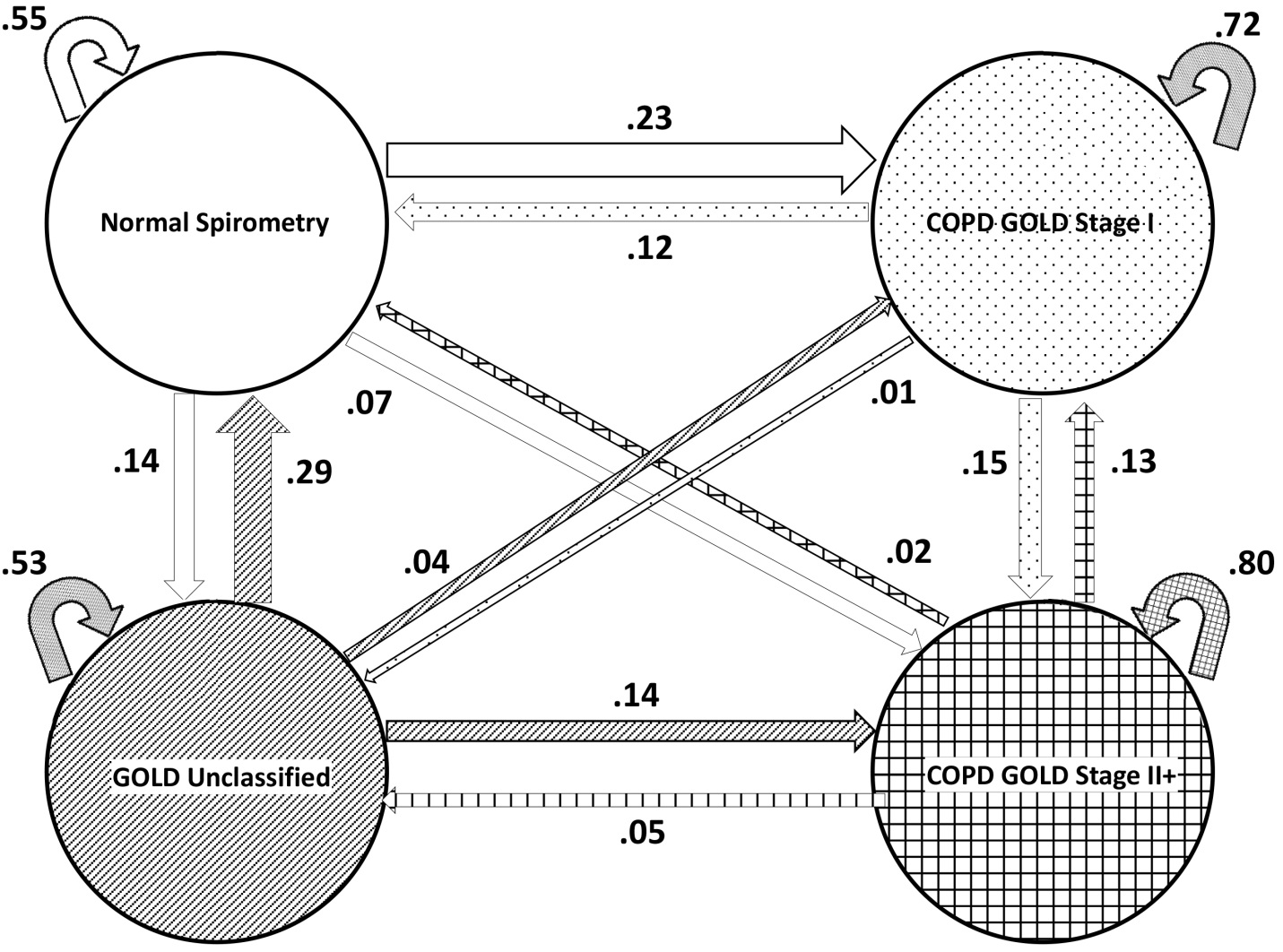


Footnote to Figure E2: Curved block arrows represents transition probabilities of staying within the same spirometric state between study examination visits at 18 month intervals. Straight arrows represent transition probabilities of change in spirometric state between study examination visits. Strength of transition probabilities is represented by the width of the straight arrow. Transition probabilities for each spirometric state are depicted in the same color as the sphere representing the spirometric state.

Supplementary Figure E3

Figure Legend E3: Study CONSORT flow diagram

**Supplementary Tables**

597 with abnormal spirometry at baseline

956 with normal spirometry at baseline

1553 eligible subjects

1895 enrolled

**Table E1:** Description of characteristics at baseline examination visit, among ‘all smokers’ and ‘smokers with normal spirometry at baseline’ separately

| Baseline characteristics | All smokers (n=1,553)  % or mean ± SD | Smokers with normal spirometry at baseline (n=956)  % or mean ± SD |
| --- | --- | --- |
| Women (%) | 78.4% | 81.9% |
| Age (mean ± SD in years) | 56 ± 9.5 | 54 ± 9.1 |
| Hispanic ethnicity (%) | 17.1% | 20.3% |
| Pack-years of smoking | 39.7 ± 20.6 | 35.7 ± 17.8 |
| (mean ± SD) |  |  |
| Current smokers (%) | 54.5% | 54.0% |
| BMI (mean ± SD in Kg/m2) | 28 ± 6.1 | 28 ± 5.5 |
| Self-reported history of hypertension (%) | 32.9% | 27.9% |
| Mean FEV1 (% predicted) | 87.6 ± 18.1 | 97.4 ± 10.7 |
| Mean FVC (% predicted) | 93.1 ± 15.0 | 97.5 ± 10.7 |
| Mean FEV1/FVC ratio | 73.4 ± 10.3 | 78.7 ± 4.5 |
| Duration of follow-up (in years) | 4.9 ± 2.5 | 5.1 ± 2.5 |
| Self-reported history of provider-diagnosed asthma | 18.2% | 13.7% |
| Presence of significant bronchodilator reversibility (%) | 5.0% | 1.8% |
| History of provider-diagnosed asthma + bronchodilator reversibility (%) | 1.5% | 0.2% |

**Table E2:** Summary of beneficial transition probabilities over approximately 5 years for spirometrically-defined disease states, described by statistically defined NHANES–III lower limits of normal for FEV_1_/FVC ratio

|  | COPD Stage I  (FEV_1_/FVC < LLN and FEV_1_ ≥ 80% predicted) | Unclassified (FEV_1_/FVC ≥ LLN and FEV_1_  < 80% predicted) | COPD Stage II+  (FEV_1_/FVC < LLN and FEV_1_ < 80% predicted) |
| --- | --- | --- | --- |
| All smokers, irrespective of spirometric disease at baseline | | | |
| All beneficial transitions | 21% | 39% | 21% |
| Resolution of disease | 21% | 35% | 4% |
| Smokers with normal spirometry at baseline* | | | |
| All beneficial transitions | 35% | 67% | 63% |
| Resolution of disease | 35% | 61% | 30% |
| Smokers with abnormal spirometry at baseline | | | |
| All beneficial transitions | 13% | 31% | 19% |
| Resolution of disease | 13% | 28% | 3% |

Note 1: A beneficial transition, our primary outcome variable, was defined by either continued maintenance of normal spirometric state or a decrease in spirometric disease state severity, including resolution, at any time during longitudinal follow-up. Resolution of disease state, our secondary outcome variable, was defined by change of spirometrically-defined diseased states to normal spirometry state at any time during longitudinal follow-up.

Note 2: * reflect transitions for new onset disease.

Note 3: A similar table using the GOLD threshold of FEV_1_/FVC ratio of ≤70% to define obstruction is presented in the main text as Table 2.

**Table E3:** Description of characteristics at baseline examination visit, among those with and without loss to follow-up after two initial visits

| **Baseline characteristics** | Those with loss to follow-up (n = 1002) | Those without loss to follow-up (n = 551) |
| --- | --- | --- |
| Women (%) | 77.8% | 78.7% |
| Age (mean ± SD in years) | 55.0 ± 9.5* | 56.7 ± 9.4 |
| Hispanic ethnicity (%) | 18.8% | 16.2% |
| Pack-years of smoking  (mean ± SD) | 40.6 ± 21.4 | 39.2 ± 20.2 |
| Current smokers (%) | 64.2%* | 49.50% |
| BMI (mean ± SD in Kg/m2) | 28.1 ± 6.3 | 28.2 ± 6.1 |
| History of hypertension (%) | 34.8% | 32.0% |
| Mean FEV1 (% predicted) | 85.0 ± 20.0* | 89.0 ± 16.9 |
| Mean FVC (% predicted) | 90.0 ± 16.0* | 90.0 ± 14.0 |
| Mean FEV1/FVC ratio | 72.9 ± 11.2 | 73.7 ± 9.8 |
| Duration of follow-up (mean ± SD in years) | 2.2 ± 1.6* | 5.6 ± 2.1 |
| Self-reported history of provider-diagnosed asthma | 19.9% | 15.2 |
| Presence of significant bronchodilator reversibility (%) | 5.6% | 4.0% |
| History of provider-diagnosed asthma + bronchodilator reversibility (%) | 1.7% | 1.1% |

Note 1: * represents p<0.05

**Table E4.** Summary of Harmful Transition Probabilities for Spirometrically-defined States at Any Time over Approximately 5 Years, Albuquerque, New Mexico, 2001-2015, Lovelace Smokers’ Cohort.

|  | Normal spirometry at any time | COPD GOLD Stage I at any time | GOLD Unclassified at any time | COPD GOLD Stage II+ at any time |
| --- | --- | --- | --- | --- |
| All smokers, irrespective of spirometric disease at baseline (n=1,553) | | | | |
| All harmful transitions | 14% | 84% | 60% | 78% |
| Smokers with normal spirometry at baseline (n=956)* | | | | |
| All harmful transitions | 11% | 75% | 37% | 37% |
| Smokers with abnormal spirometry at baseline (n=597) | | | | |
| All harmful transitions | 23% | 88% | 67% | 80% |

Abbreviations: COPD: Chronic Obstructive Pulmonary Disease; GOLD: Global Initiative for Chronic Obstructive Lung Disease

^1^ A Harmful transition was defined by either increase in spirometric state severity, or continued maintenance of COPD GOLD Stage II+ state at any time during longitudinal follow-up.

^2^ * reflect transitions for new onset disease.

**Table E5.** Distribution of subjects (with at least 4 visits) by baseline spirometric state, displaying different disease trajectories over 5 years.

| Characteristic | Smokers with normal spirometry (n=575) | COPD GOLD stage I (n=81) | GOLD Unclassified (n=111) | COPD GOLD stage II+ (n=145) |
| --- | --- | --- | --- | --- |
| No Change in Disease State | 72.5% | 51.9% | 18% | 57.2% |
| Uniform Worsening of Disease State | 13.7% | 7.4% | 9.9% | 0% |
| Uniform Improvement of Disease State | 0% | 4.9% | 29.7% | 21.4% |
| Oscillation between Disease States | 13.7% | 35.8% | 42.3% | 21.4% |

Abbreviations: COPD: Chronic Obstructive Pulmonary Disease; GOLD: Global Initiative for Chronic Obstructive Lung Disease

Note 1: In order to depict oscillations in states, we examined various disease trajectories using a non-Markov approach. Since this approach has different assumptions, different time periods of observations, and different analytic technique than the approach in the main text, this information is only provided in the supplement. This data demonstrate that oscillations are most common in GOLD Unclassified state and least common in smokers with normal spirometry.

**Supplemental References**

1. Standardization of spirometry, 1994 update. American Thoracic Society. *American journal of respiratory and critical care medicine* 1995;152:1107-1136.

2. Pellegrino R, Decramer M, van Schayck CP, Dekhuijzen PN, Troosters T, van Herwaarden C, Olivieri D, Del Donno M, De Backer W, Lankhorst I, Ardia A. Quality control of spirometry: A lesson from the Broncus trial. *Eur Respir J* 2005;26:1104-1109.

3. Hankinson JL, Odencrantz JR, Fedan KB. Spirometric reference values from a sample of the general u.S. Population. *American journal of respiratory and critical care medicine* 1999;159:179-187.

4. Jones PW, Quirk FH, Baveystock CM, Littlejohns P. A self-complete measure of health status for chronic airflow limitation. The St. George's respiratory questionnaire. *The American review of respiratory disease* 1992;145:1321-1327.
